# Real-World Effectiveness of Simeprevir-containing Regimens Among Patients With Chronic Hepatitis C Virus: The SONET Study

**DOI:** 10.1093/ofid/ofw258

**Published:** 2016-12-26

**Authors:** Imtiaz Alam, Kimberley Brown, Cynthia Donovan, Jamie Forlenza, Kris Lauwers, Mitchell A. Mah’moud, Richard Manch, Smruti R. Mohanty, Avinash Prabhakar, Robert Reindollar, Ralph DeMasi, Jihad Slim, Neeta Tandon, Shirley Villadiego, Susanna Naggie

**Affiliations:** 1 Austin Hepatitis Center, Austin, Texas; 2 Janssen Scientific Affairs, Titusville, New Jersey; 3 Janssen Research & Development, Beerse, Belgium; 4 Department of Medicine, Duke University School of Medicine/Department of Gastroenterology, Boice-Willis Clinic, Rocky Mount, North Carolina; 5 St. Joseph’s Hospital and Medical Center, Phoenix, Arizona; 6 New York Methodist Hospital, Brooklyn, New York; 7 Piedmont Healthcare, Gastroenterology and Hepatology, Statesville, North Carolina; 8 Department of Infectious Disease, Saint Michael’s Medical Center, Newark, New Jersey; 9 Durham VA Medical Center/Department of Medicine, Duke University School of Medicine, Durham, North Carolina

**Keywords:** hepatitis C virus, real-world, simeprevir

## Abstract

**Background:**

The Simeprevir ObservatioNal Effectiveness across practice seTtings (SONET) study evaluated the real-world effectiveness of simeprevir-based treatment for hepatitis C virus (HCV) infection.

**Methods:**

The SONET study was a phase 4, prospective, observational, United States–based study enrolling patients ≥18 years of age with chronic genotype 1 HCV infection. The primary endpoint was the proportion of patients who achieved sustained virologic response 12 weeks after the end of treatment (SVR12), defined as HCV ribonucleic acid undetectable ≥12 weeks after the end of all HCV treatments.

**Results:**

Of 315 patients (intent-to-treat [ITT] population), 275 (87.3%) completed the study. Overall, 291 were treated with simeprevir + sofosbuvir, 17 with simeprevir + sofosbuvir + ribavirin, and 7 with simeprevir + peginterferon + ribavirin. The majority of patients were male (63.2%) and white (60.6%); median age was 58 years, 71.7% had genotype/subtype 1a, and 39.4% had cirrhosis. The SVR12 was achieved by 81.2% (255 of 314) of ITT patients (analysis excluded 1 patient who completed the study but was missing SVR12 data); 2 had viral breakthrough and 18 had viral relapse. The SVR12 was achieved by 92.4% (255 of 276) of patients in the modified ITT (mITT) population, which excluded patients who discontinued treatment for nonvirologic reasons before the SVR12 time point or were missing SVR12 assessment data. Among mITT patients, higher SVR12 rates were associated with factors including age ≥65 years, non-Hispanic/Latino ethnicity, and employment status, but not genotype/subtype nor presence of cirrhosis. Simeprevir-based treatment was well tolerated; no serious adverse events were considered related to simeprevir.

**Conclusions:**

In the real-world setting, simeprevir + sofosbuvir treatment was common and 92% of mITT patients achieved SVR12. Simeprevir-based treatment was effective and well tolerated in this cohort, including patients with cirrhosis.

Chronic hepatitis C virus (HCV) infection is a global health concern and a leading cause of liver disease [1]. The introduction of direct-acting antiviral (DAA) agents has expanded therapeutic options with improved safety and efficacy profiles [2, 3]. This rapidly changing treatment landscape necessitates evaluation of new agents in real-world settings to understand the potential of the regimens [4]. Several studies have reported real-world outcomes for DAA regimens; examples include analyses of HCV-TARGET, TRIO, and US Veterans Administration databases, as well as studies involving more limited populations [5–11].

The combination of simeprevir, a once-daily NS3/4A protease inhibitor, and sofosbuvir, a once-daily NS5B polymerase inhibitor, with or without ribavirin, is approved in the United States for the treatment of genotype 1 HCV infection, based on the phase 2 COSMOS study [12]. The phase 3 OPTIMIST-1 and OPTIMIST-2 studies have further supported the safety and efficacy of this combination, demonstrating high rates of sustained virologic response with a 12-week treatment regimen in patients without or with cirrhosis [13, 14]. In patients with cirrhosis, 24 weeks of therapy is recommended [15].

Data observed in routine clinical practice can vary significantly from results obtained from clinical studies. The Simeprevir ObservatioNal Effectiveness across practice seTtings (SONET) study was conducted to provide real-world data on simeprevir-based regimens among diverse patients representative of the US population.

## PATIENTS AND METHODS

### Study Design

The SONET study was a phase 4, multicenter, observational study (ClinicalTrials.gov Identifier: NCT02103699) conducted from February 2014 through November 2015 at 37 sites in the United States (31 sites enrolled patients). The primary objective was to measure the effectiveness of simeprevir-containing regimens for patients with chronic HCV infection. In addition, patient and practice setting characteristics, virologic response rates and factors associated with virologic response, and safety outcomes are described.

Patients were enrolled from a variety of practice settings, including academic medical centers, private practice clinics, and integrated delivery healthcare systems; some sites had features of >1 type of practice setting and were thus characterized as “>1 setting.” Patients were consecutively screened for eligibility by the participating healthcare provider (HCP) to ensure a population representative of routine clinical practice and reduce selection bias. Eligible patients were offered enrollment; those who entered the study received their simeprevir-containing HCV treatment regimen as per routine clinical practice. All treatment decisions and assessments were made at the discretion of the HCP.

Data from patients’ medical records were collected throughout treatment and until posttreatment assessment of sustained virologic response 12 weeks after the end of treatment (SVR12). Posttreatment assessments were performed according to local standards of care; available data for visits at 4 and 12 weeks after treatment completion were recorded. Patient-completed surveys provided additional information (see Supplemental Materials).

Patients provided written informed consent, and the study was conducted in accordance with the ethical principles that have their origin in the Declaration of Helsinki and are consistent with Good Clinical Practices and applicable regulatory requirements. The study protocol was reviewed by institutional review boards.

### Patients

Eligible patients were ≥18 years of age with chronic HCV genotype 1 infection, an HCV ribonucleic acid (RNA) test result above the lower limit of quantification (LLOQ), and an HCP decision to treat with simeprevir-based therapy (including patients prescribed simeprevir-based therapy at the time of enrollment and those receiving simeprevir-based therapy for ≤28 days at the time of enrollment). For treatment-experienced patients, prior HCV treatment (peginterferon, ribavirin) was completed >3 months before initiation of simeprevir-based therapy. Patients with previous use of DAA therapy were excluded.

Cirrhosis status was based on medical record reviews; if cirrhosis was present, the type of assessment (biopsy, imaging, ultrasound, elastography, noninvasive markers) was documented. Hepatic decompensation was defined as the presence of esophageal and/or gastric varices, ascites, and/or hepatic encephalopathy. See Supplemental Materials for additional information.

### Effectiveness Assessments

The primary endpoint was the proportion of patients who achieved SVR12, defined as HCV RNA undetectable ≥12 weeks after the end of all HCV treatments. Secondary endpoints included the proportions of patients who had sustained virologic response 4 weeks after the end of treatment (SVR4) and rapid virologic response (RVR; see Supplemental Materials for definitions), as well as viral breakthrough (defined as a confirmed >1.0 log_10_ IU/mL increase in HCV RNA level from the lowest level reached, or confirmed HCV RNA >100 IU/mL in patients whose HCV RNA had previously been below the LLOQ while on treatment) and viral relapse (defined as detectable HCV RNA after achieving undetectable HCV RNA at the end of treatment). Nonresponders were defined as patients who never reached HCV RNA below the LLOQ undetectable. The proportion of patients who achieved SVR12 by subgroup was also evaluated, and an exploratory logistic regression analysis was used to identify prognostic factors for SVR12 achievement. Hepatitis C virus RNA testing was performed in local laboratories (Supplemental Materials).

### Safety Assessments

All adverse events (AEs) after exposure to simeprevir, and for 30 days after a patient’s last dose of simeprevir, were documented by the HCP (Supplemental Materials).

### Statistical Analyses

Statistical analyses were performed using SAS^®^ version 9.1.3 (SAS Institute Inc., Cary, NC). No imputation rules for missing data were implemented; data were summarized as collected in the study. A total sample size of approximately 300 patients was planned, which would allow the SVR12 rate to be estimated (z-distribution) with a 2-sided 95% confidence interval (CI) and precision of 5.35% on each side of the estimate of response (hypothesized to be 70%; 95% CI, 64.65–75.35) [16, 17].

All analyses (except subgroup and prognostic factor analyses) were performed based on the intent-to-treat (ITT) population, defined as all patients who were enrolled and received ≥1 dose of simeprevir. Subgroup analyses were conducted based on the modified ITT (mITT) population, defined as all patients in the ITT population excluding those who discontinued for nonvirologic reasons before the SVR12 time point, or for whom SVR12 assessment data were missing. Exploratory, prognostic factor analyses were conducted for patients in the mITT population who were in the simeprevir + sofosbuvir treatment group.

Study endpoints were assessed using summary statistics (Supplemental Materials). No formal hypothesis was tested. The proportion who achieved SVR12 (primary endpoint) was tabulated along with the 2-sided 95% CI and based on a snapshot approach, in which viral load at follow-up Week 12 takes precedence over earlier outcomes. Patients who discontinued during treatment, or during the follow-up phase and had no HCV viral load data in the SVR12 time window, were regarded as not having achieved SVR12. Rapid virologic response was assessed for patients who had a study visit at the RVR time point. The SVR4 was assessed for patients who had a study visit at the SVR4 time point and those who were missing the SVR4 visit but had previously failed or had SVR12 assessed.

For the prognostic factor analysis, each covariate was evaluated using univariate logistic regression models, followed by initial multivariate regression analyses forcing in all variables with *P* < .90. Starting with all variables with *P* < .15 from initial multivariate analyses, a backward stepwise selection process was used with a stay selection criteria of *P* < .15 to arrive at the final model. Covariates that were missing more than 20% of the values were not considered.

## RESULTS

### Study Population

#### Patients

**Figure 1.**
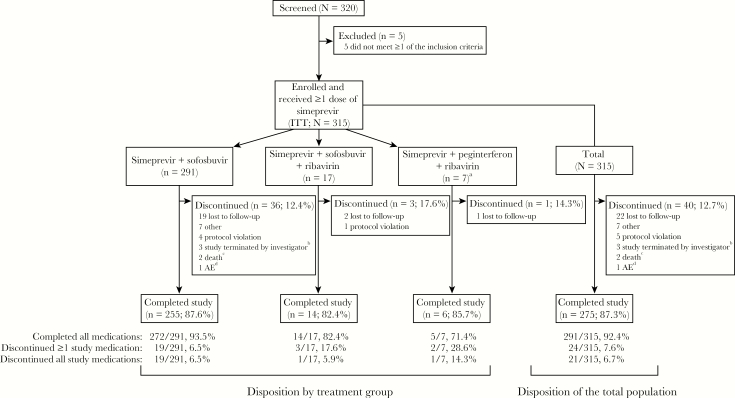
Patient disposition. ^a^Data for the simeprevir + peginterferon + ribavirin group are not reported due to the small sample size; however, data for these patients are included in the total population. ^b^Patients were discontinued when the investigator decided to no longer participate in the study. ^c^Both deaths occurred >30 days after the last dose of study medication and were considered unrelated to simeprevir. ^d^Adverse event (AE) was grade 3 thrombocytopenia and was considered very likely related to simeprevir and sofosbuvir. ITT, intent to treat.

Of 320 patients screened, 315 enrolled in the study and received ≥1 dose of simeprevir (ITT population; Figure 1). Three regimens were prescribed: 291 of 315 (92.4%) patients received simeprevir + sofosbuvir, 17 of 315 (5.4%) received simeprevir + sofosbuvir + ribavirin, and 7 of 315 (2.2%) received simeprevir + peginterferon + ribavirin. Findings are not reported for the simeprevir + peginterferon + ribavirin group due to the small sample size; however, these data are included in analyses for the total population.

Demographic characteristics were generally similar across treatment groups (Table 1). Overall, 124 of 315 (39.4%) patients had cirrhosis and 41 of 315 (13.0%) had hepatic decompensation, whereas 95 of 315 (30.2%) were treatment-experienced. Only 22 patients had data available for assessment of NS3 Q80K polymorphism; 7 of 22 (31.8%) had Q80K. Patient-completed survey results are summarized in the Supplemental Materials.

**Table 1.** Baseline Demographic and Disease Characteristics

	Simeprevir + Sofosbuvir (n = 291)	Simeprevir + Sofosbuvir + Ribavirin (n = 17)	Total (N = 315)^a^
Demographic characteristics			
Age, median (range), year	58.0 (18–82)	59.0 (42–68)	58.0 (18–82)
Gender, n (%)			
Female	111 (38.1)	3 (17.6)	116 (36.8)
Male	180 (61.9)	14 (82.4)	199 (63.2)
Race, n (%)^b^			
White	174 (59.8)	11 (64.7)	191 (60.6)
Black/African American	102 (35.1)	6 (35.3)	108 (34.3)
Other^c^	15 (5.2)	0	16 (5.1)
Ethnicity, n (%)^b^			
Hispanic/Latino	45 (15.5)	0	46 (14.6)
Not Hispanic/Latino	241 (82.8)	17 (100)	264 (83.8)
Other^d^	5 (1.7)	0	5 (1.6)
BMI, median (IQR), kg/m^2e^	28.3 (24.6–32.2)	28.5 (24.7–30.1)	28.1 (24.6–32.1)
Disease characteristics			
HCV RNA level, median (range), log_10_ IU/mL	6.3 (1.4–7.6)	6.2 (5.6–7.8)	6.2 (1.4–7.8)
HCV genotype/subtype, n (%)			
1a	209 (71.8)	13 (76.5)	226 (71.7)
1b	62 (21.3)	3 (17.6)	67 (21.3)
Indeterminate/other	20 (6.9)	1 (5.9)	22 (7.0)
Presence of cirrhosis, n (%)	116 (39.9)	6 (35.3)	124 (39.4)
Hepatic decompensation, n (%)	40 (13.7)	1 (5.9)	41 (13.0)
Esophageal and/or gastric varices, n (%)			
History, not active	13 (4.5)	0	13 (4.1)
Active	27 (9.3)	0	27 (8.6)
Ascites, n (%)			
History, not active	7 (2.4)	0	7 (2.2)
Active	15 (5.2)	1 (5.9)	16 (5.1)
Hepatic encephalopathy, n (%)			
History, not active	1 (0.3)	0	1 (0.3)
Active	16 (5.5)	0	16 (5.1)
Calculated MELD score category, n (%)^f^			
≤10	90 (76.9)	6 (100)	96 (78.0)
≥11 to ≤18	26 (22.2)	0	26 (21.1)
≥19 to ≤24	0	0	0
≥25	1 (0.9)	0	1 (0.8)
HIV coinfection, n (%)	23 (7.9)	0	23 (7.3)

Abbreviations: BMI, body mass index; IQR, interquartile range; HCV, hepatitis C virus; HIV, human immunodeficiency virus; MELD, Model for End-stage Liver Disease; RNA, ribonucleic acid.

^a^Includes 7 patients treated with simeprevir + peginterferon + ribavirin.

^b^Race and ethnicity data were obtained from patients’ medical records.

^c^“Other” includes Asian, American Indian or Alaska Native, other, unknown, and not reported.

^d^Other includes unknown and not reported.

^e^n = 288 for the simeprevir + sofosbuvir group; total N = 312.

^f^n = 117 for the simeprevir + sofosbuvir group; n = 6 for the simeprevir + sofosbuvir + ribavirin group; total N = 123.

#### Disposition and Treatment Duration

Treatment was completed by 291 of 315 (92.4%) patients, and the study was completed by 275 of 315 (87.3%) patients (Figure 1). The most common reason for discontinuation was loss to follow-up (22 of 40; 55.0%).

In total, 258 of 315 (81.9%) patients received 12 weeks of treatment; for patients without cirrhosis, 170 of 191 (89.0%) received treatment for 12 weeks. Among patients with cirrhosis, 88 of 124 (71.0%) received 12 weeks of treatment and 25 of 124 (20.2%) received 24 weeks. See Supplemental Materials for additional information.

#### Practice Settings

Overall, 63 of 315 (20.0%) patients received care at academic medical centers, 199 of 315 (63.2%) at private practice clinics, 31 of 315 (9.8%) at integrated delivery healthcare systems, and 22 of 315 (7.0%) at clinics that were characterized as >1 setting.

### Effectiveness

#### Virologic Response

Among patients in the ITT population, RVR was achieved by 151 of 243 (62.1%) in the simeprevir + sofosbuvir group and 12 of 15 (80.0%) in the simeprevir + sofosbuvir + ribavirin group (169 of 264 [64.0%] overall). The SVR4 was achieved by 236 of 290 (81.4%) patients in the simeprevir + sofosbuvir group and 14 of 16 (87.5%) in the simeprevir + sofosbuvir + ribavirin group (254 of 313 [81.2%] overall).

**Figure 2.**
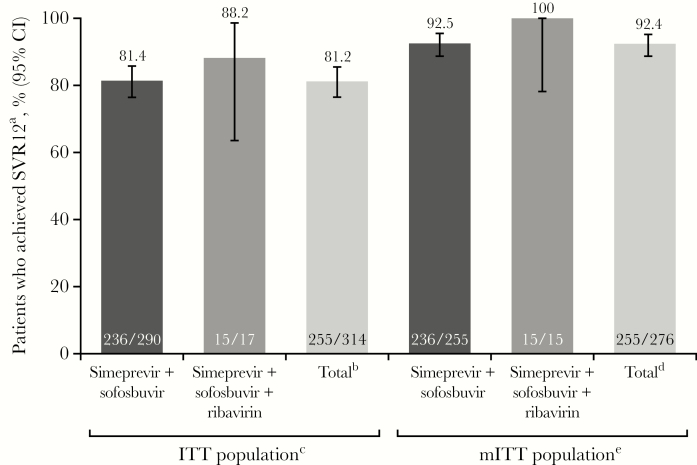
Achievement of SVR12. ^a^Sustained virologic response 12 weeks after the end of treatment (SVR12) analysis was based on a snapshot approach. ^b^Includes 7 patients treated with simeprevir + peginterferon + ribavirin. ^c^Excludes 1 patient who completed treatment and the study but was missing data for the SVR12 time point. ^d^Includes 6 patients treated with simeprevir + peginterferon + ribavirin. ^e^The modified intent-to-treat (mITT) population includes patients in the intent-to-treat (ITT) population excluding those who discontinued for nonvirologic reasons before the SVR12 time point, or with missing SVR12 assessment data (38 patients and 1 patient, respectively). CI, confidence interval.

In the ITT population, SVR12 was achieved by 255 of 314 (81.2%) patients (Figure 2; 98 of 123 [79.7%] patients with cirrhosis); the analysis excluded 1 patient who completed treatment and the study but was missing data for the SVR12 time point (see Supplemental Materials for additional information). Of those patients who did not achieve SVR12, 4 of 59 (6.8%) had no response and 38 of 59 (64.4%) discontinued before the SVR12 time point; patients who had virologic failure are described below. The mITT population excluded 39 of 315 (12.4%) patients in the ITT population, primarily due to study discontinuation before the SVR12 time point (38 of 39; 97.4%); the remaining patient (1 of 39; 2.6%) was excluded due to missing data at the SVR12 time point. Among mITT patients, 255 of 276 (92.4%) achieved SVR12, including 98 of 107 (91.6%) patients with cirrhosis and 15 of 15 (100%) of those who received simeprevir + sofosbuvir + ribavirin.

#### Factors Associated With SVR12 Achievement

The SVR12 rates across subgroups in the mITT population are summarized in Supplemental Table 1. Large (>5%) differences were observed for several factors; higher SVR12 rates were achieved by patients who were older (≥65 years, 100%) versus younger (≤45 years, 88.9%; >45 to <65 years, 91.0%), non-Hispanic/Latino (93.5%) versus Hispanic/Latino (85.7%), black/African American female (97.1%) versus white female (91.2%) and versus black/African American male (90.0%), had a body mass index (BMI) of ≥25 to <30 kg/m^2^ (95.5%) versus ≥30 kg/m^2^ (90.1%), and were not coinfected with human immunodeficiency virus (HIV; 93.7%) versus coinfected with HIV (77.3%). The SVR12 rates also showed variations (>5%) based on socioeconomic factors; higher SVR12 rates were observed for patients with an employment status of retired (96.0%) versus self-employed (81.3%), moved at least once in the past year (100%) versus lived in one place the whole time (92.2%), and spent $1–$25 on out-of-pocket medication costs monthly (93.3%) versus spent $26–$50 (87.0%). Higher rates of SVR12 were also observed for patients who had earlier virologic response: those who achieved RVR (95.5%) versus those who did not (85.5%), and those who achieved virologic response at the end of treatment (93.9%) versus those who did not (80.0%).

Results of the SVR12 prognostic factor analysis showed that non-Hispanic/Latino ethnicity, older age (by decade), lower BMI, and absence of HIV coinfection were each associated with SVR12 achievement in both univariate and multivariate analyses (*P* < .1; Supplemental Table 2). Only a small number of patients were coinfected with HIV overall (23 of 315; 7.3%) (Table 1), but this factor was most statistically significantly associated with SVR12 achievement (*P* < .01) and was also identified in the subgroup analysis as a factor associated with treatment outcome. Annual income and out-of-pocket medication costs were not considered as covariates in the prognostic factor analysis due to missing values; other excluded variables were treatment duration (due to a high correlation with cirrhosis) and living situation (due to a stable living situation for most patients).

#### Virologic Failure

In total, 2 of 315 (0.6%) patients experienced viral breakthrough: 1 in the simeprevir + sofosbuvir group (per protocol definitions, this patient was a breakthrough with detectable [but below the LLOQ] HCV RNA at on-treatment Week 12, and also achieved SVR12 with a later confirmed undetectable HCV RNA at posttreatment Week 24) and 1 in the simeprevir + peginterferon + ribavirin group (viral breakthrough was detected at Week 24 [end of treatment]). Overall, 18 of 315 (5.7%) patients had viral relapse: 17 in the simeprevir + sofosbuvir group and 1 in the simeprevir + peginterferon + ribavirin group (Table 2). Eight of the 18 (44.4%) patients who relapsed had cirrhosis (5 received 12 weeks of simeprevir treatment; 3 received ≥24 weeks of simeprevir treatment), 6 of 18 (33.3%) were treatment-experienced, 11 of 18 (61.1%) had HCV genotype/subtype 1a (neither of the 2 patients with available data had the NS3 Q80K polymorphism), and 6 of 18 (33.3%) had genotype/subtype 1b (genotype/subtype was indeterminate for 1 patient). One patient (HCV genotype/subtype 1b, treatment-naïve, without cirrhosis) who achieved SVR12 was also considered a relapse per protocol definitions. This patient (who was included in the above group of 18 relapsers) had undetectable HCV RNA at Week 12, which became detectable at posttreatment Week 16 and was undetectable at posttreatment Week 24; it was not confirmed why HCV RNA was detectable at Week 16, and there was no report of additional HCV treatment after Week 16. All patients with viral relapse completed the assigned treatment as planned, except 1 in the simeprevir + sofosbuvir group who discontinued all treatment after 8 weeks (patient decision [too many pills]).

**Table 2.** Cirrhosis Status and Treatment Experience for Patients Who Had Viral Relapse

Patient Group	Simeprevir + Sofosbuvir	Simeprevir + Sofosbuvir + Ribavirin	Total^a^
No cirrhosis; treatment-naïve	6	0	7^b^
No cirrhosis; treatment-experienced	3	0	3
Cirrhosis; treatment-naïve	5	0	5
Cirrhosis; treatment-experienced	3	0	3

Abbreviations: SVR12, sustained virologic response 12 weeks after the end of treatment.

^a^Includes 7 patients treated with simeprevir + peginterferon + ribavirin.

^b^Includes 1 patient in the simeprevir + peginterferon + ribavirin group; for this patient, viral breakthrough was detected at Week 24 of treatment (end of treatment) and SVR12 was not achieved.

### Safety

Of 315 patients in the ITT population, 178 (56.5%) had ≥1 AE during the study; the most common AEs were headache, fatigue, and nausea (Table 3). Ninety-six (30.5%) patients had an AE that the HCP considered at least possibly related to simeprevir. Twenty-six (8.3%) patients had a serious AE; none were considered possibly related to simeprevir. Most AEs were grade 1 or 2; 24 (7.6%) patients had a grade 3 AE, and 1 (0.3%) patient had a grade 4 AE (accidental overdose). Two (0.6%) patients had a fatal AE that occurred during the posttreatment period; both (acute intracerebral hemorrhage and cerebrovascular accident) were grade 3 in severity and not considered related to simeprevir treatment.

**Table 3.** Summary of Safety During the Study Period

AE Parameter	Simeprevir + Sofosbuvir	Simeprevir + Sofosbuvir + Ribavirin	Total^a^
All patients, n (%)	n = 291	n = 17	N = 315
Any AE	159 (54.6)	13 (76.5)	178 (56.5)
Any serious AE	24 (8.2)	2 (11.8)	26 (8.3)
Any AE at least possibly related to simeprevir	91 (31.3)	5 (29.4)	96 (30.5)
Grade ≥2	28 (9.6)	2 (11.8)	30 (9.5)
Any AE leading to permanent stop of ≥1 study medication^b^	3 (1.0)	1 (5.9)	4 (1.3)
Any fatal AE	2 (0.7)	0	2 (0.6)
Most common (>3% of patients) AEs			
Headache	39 (13.4)	2 (11.8)	41 (13.0)
Nausea	34 (11.7)	1 (5.9)	35 (11.1)
Fatigue	32 (11.0)	6 (35.3)	40 (12.7)
Insomnia	15 (5.2)	4 (23.5)	21 (6.7)
Rash	11 (3.8)	1 (5.9)	14 (4.4)
Abdominal pain	10 (3.4)	0	10 (3.2)
Diarrhea	10 (3.4)	0	10 (3.2)
Dyspnea	8 (2.7)	3 (17.6)	11 (3.5)
Anemia	7 (2.4)	4 (23.5)	13 (4.1)
Patients with cirrhosis, n (%)	n = 116	n = 6	n = 124
Any AE	68 (58.6)	6 (100)	76 (61.3)
Any serious AE	15 (12.9)	2 (33.3)	17 (13.7)
Any AE at least possibly related to simeprevir	38 (32.8)	2 (33.3)	40 (32.3)
Grade ≥2	12 (10.3)	0	12 (9.7)
Patients without cirrhosis, n (%)	n = 175	n = 11	n = 191
Any AE	91 (52.0)	7 (63.6)	102 (53.4)
Any serious AE	9 (5.1)	0	9 (4.7)
Any AE at least possibly related to simeprevir	53 (30.3)	3 (27.3)	56 (29.3)
Grade ≥2	16 (9.1)	2 (18.2)	18 (9.4)

Abbreviation: AE, adverse event.

^a^Includes 7 patients treated with simeprevir + peginterferon + ribavirin.

^b^The 3 patients in the simeprevir + sofosbuvir group each discontinued both study medications; AEs leading to discontinuation were chronic kidney disease (serious AE, not related to simeprevir), renal impairment (not serious, not related to simeprevir), and thrombocytopenia (very likely related to both simeprevir and sofosbuvir). The 1 patient in the simeprevir + sofosbuvir + ribavirin group discontinued ribavirin only due to anemia (very likely related to ribavirin).

Adverse events were more common in patients with cirrhosis than in those without and also in those treated with ribavirin versus without (Table 3); however, the proportion of patients who had an AE considered possibly related to simeprevir was similar across the subgroups.

A decrease in median hemoglobin levels was observed in the ribavirin-containing treatment groups during the first 4 weeks of treatment; values remained relatively stable through the end of treatment (see Supplemental Materials for additional laboratory parameters).

## DISCUSSION

The SONET study was conducted to provide generalizable, real-world data describing simeprevir outcomes among diverse patients in the United States; SONET also examined patient characteristics, virologic response rates and factors associated with virologic response, and safety outcomes of patients who receive simeprevir in various practice settings. This study demonstrated that simeprevir-based treatment was well tolerated and that high SVR12 rates (>90%) can be achieved in the real world, consistent with other studies [5–11], and also underscores the challenge of patient follow-up outside of clinical trials.

The most common treatment regimen was simeprevir + sofosbuvir (n = 255); few patients (n = 17) received concomitant ribavirin. Overall, SVR12 was achieved by 81.2% of patients in the ITT population; this rate was affected by study discontinuations before the SVR12 time point (primarily due to loss to follow-up). The SVR12 rate for mITT patients was 92.4%, highlighting the efficacy of DAA therapy in patients who were able to complete therapy and remain engaged in care. In these patients, SVR12 rates were similar to those observed in the COSMOS, OPTIMIST-1, and OPTIMIST-2 clinical studies [12–14]. Although a higher proportion of patients on simeprevir + sofosbuvir + ribavirin achieved SVR12 compared with those on simeprevir + sofosbuvir, the number of patients treated with ribavirin was relatively small, and the impact of ribavirin on SVR12 rates could not be formally evaluated.

Previous results have been mixed in terms of a relationship between HCV genotype/subtype and SVR12 rates in real-world settings [5, 18]. In SONET, subgroup analyses did not show an association, although genotype/subtype 1a was more common than genotype/subtype 1b among patients with viral relapse (11 vs 6 patients, respectively). Q80K polymorphism testing was uncommon and not associated with treatment outcome. Other historic predictors of virologic response, including cirrhosis and hepatic decompensation, were also not associated with outcomes. However, SONET did reveal factors associated with achieving SVR12, including demographic characteristics, socioeconomic factors, and earlier virologic response. This study did not evaluate how these factors may contribute to SVR12 achievement, and it is possible that the effects of some characteristics are mediated by other causes. For example, financial stress may influence treatment adherence, which may affect outcomes [19, 20]. Similarly, absence of HIV coinfection was the independent variable with the strongest association with SVR12 achievement in the prognostic factor analysis (*P* < .01), but due to the small number of patients coinfected with HIV, it was not possible to determine whether HIV status was associated with other prognostic factors that, in turn, were associated with lower SVR12 rates. Moreover, this finding should be considered in the greater context of available data; an association between HIV coinfection and response to treatment with DAA therapy has not been reported in other studies [21, 22].

The SONET study highlights the challenge of using an ITT analysis for observational studies because a considerable number of patients were lost to follow-up and considered to be nonresponders per ITT analysis guidance from the US Food and Drug Administration (FDA) [23]. In addition, a relatively small proportion of patients with cirrhosis (20.2%) received the recommended 24-week treatment regimen [15]; this is likely related to the timing of FDA approval of the regimen, which occurred while SONET was already in progress. The proportion of patients who had cirrhosis (39.4%) was higher than that seen in most clinical trials, suggesting that clinicians in the real world are treating patients with more advanced disease than those included in clinical studies. Also of note, healthcare resource utilization measures were assessed as a secondary endpoint; however, the data captured lacked specificity and completeness, making them difficult to interpret, and, as a result, they were not included here.

Another challenge during SONET was the difficulty in recruiting HCPs in some practice settings of interest, which may have limited population diversity. There were 3 sites initially identified as community health centers that were invited to be included in the study, but 2 of these sites were later reclassified and 1 did not enroll any patients. An opiate dependency treatment center was invited to be included in the study but ultimately declined to participate. A greater diversity of practice settings would have been useful to further evaluate implementation of HCV treatment regimens in the community, as opposed to a clinical trial setting. Although other real-world studies of DAA-based regimens have been reported [5–11], the associations between treatment outcome and practice setting characteristics, as well as patients’ socioeconomic status, have not been well defined. There are many factors that can influence treatment uptake and adherence, including patients’ interactions with staff members at treatment facilities and socioeconomic status characteristics [24, 25]. This study aimed to assess several such factors; however, limitations such as missing data and correlations between covariates prevented the inclusion of some such factors in the analyses.

It is notable that, since the SONET study began in 2014, tremendous progress has been made in the treatment of HCV infection [2–4]. Simeprevir was initially indicated for combination use with peginterferon and ribavirin, and it has since been approved for use in combination with sofosbuvir [15]. Additional agents continue to be approved, increasing treatment options with 6 different DAA combinations recommended for the treatment of genotype 1 infection based on HCV guidance from the American Association for the Study of Liver Diseases and Infectious Diseases Society of America [2]. Therefore, the data reported here remain important to describe the use of simeprevir in combination with sofosbuvir to those clinicians who require access to this treatment regimen and to report on the effectiveness of DAA combination therapies in the real world.

## CONCLUSIONS

In summary, SONET demonstrated the effectiveness and safety of simeprevir-based HCV therapies in real-world settings, and it is consistent with findings observed in the clinical trial setting [12–14] and previous real-world studies [5–11] of simeprevir + sofosbuvir treatment. This study suggests that few factors negatively impact the effectiveness of potent HCV treatment regimens such as simeprevir + sofosbuvir.

## Supplementary Data

Supplementary materials are available at *Open Forum Infectious Diseases* online. Consisting of data provided by the authors to benefit the reader, the posted materials are not copyedited and are the sole responsibility of the authors, so questions or comments should be addressed to the corresponding author.

## Supplementary Material

ofw258_suppl_supplemental_materials
