# Supplementary material for: Real-World Effectiveness of Simeprevir-containing Regimens Among Patients With Chronic Hepatitis C Virus: The SONET Study
Source: Open Forum Infect Dis. 2016 Dec 26;4(1):ofw258. doi: 10.1093/ofid/ofw258 (PMC5413999; doi:10.1093/ofid/ofw258)
Supplement: ofw258_suppl_supplemental_materials [file ofw258_suppl_supplemental_materials.docx]

**Supplemental Materials**

**Methods**

*Patients*

Additional key exclusion criteria were any absolute contraindication to any component of prescribed hepatitis C virus (HCV) treatment per prescribing information, enrollment in an interventional study, and use of an investigational drug within 30 days of initiation of simeprevir-based therapy.

Data were collected throughout the HCV treatment course and until posttreatment assessment of sustained virologic response 12 weeks after the end of treatment (SVR12; ie, the observational phase). Study sites entered available on-treatment data from the patient’s medical records (electronic medical records, charts) at least every 4 weeks for the first 12 weeks of HCV treatment; after Week 12, data were entered every 12 weeks while the patient was still on treatment, and at the actual end of all HCV treatment. Posttreatment assessments were performed according to the local standard of care; available data for visits at 4 and 12 weeks after treatment completion were recorded. Collected information included clinical laboratory evaluations that were performed as part of routine clinical care.

*Effectiveness Assessments*

Sustained virologic response 4 weeks after the end of treatment (SVR4) was defined as HCV RNA undetectable ≥4 to ≤8 weeks after the end of all HCV treatments. Rapid virologic response (RVR) was defined as HCV RNA undetectable at treatment Week 4. HCV RNA testing was performed at local laboratories, which varied in RNA measurement methodology and thus varied in lower limit of quantification (LLOQ) cutoff values. However, the vast majority (~90%) of patients had HCV RNA assayed using the same LLOQ (15 IU/mL); the second and third most common LLOQ cutoffs were 43 IU/mL (~4% of patients) and 615 IU/mL (~2% of patients). All qualitative and quantitative HCV RNA levels that were reported in the patient’s medical record during the prospective observational data collection phase were recorded in the patient’s electronic case report form.

*Patient-completed Surveys*

Patients were asked to complete surveys on paper forms at the time of enrollment and at the first visit after all HCV treatments were completed or discontinued; this could occur at the patient visit when end-of-treatment data collection occurred. If there was no visit within 16 weeks after all HCV treatments were completed or discontinued, the patient was contacted and provided with instructions on how to complete the survey (eg, via onsite, mailed, or emailed paper survey).

*Safety Assessments*

Healthcare providers recorded their assessment concerning the relationship between the adverse event (AE) and any HCV treatment. AE severity was assessed using the World Health Organization toxicity grading scale [1]. Available clinical laboratory results that were obtained as part of the patient’s usual standard of care were collected. Hematology and chemistry parameter results were documented during baseline data collection at enrollment and during the observational phase.

*Statistical Analyses*

All study endpoints were assessed using summary statistics. Continuous variables were summarized using descriptive statistics; categorical variables were summarized using number and percentage of patients.

**Results**

*Study Population*

Treatment duration

In total, 15/315 (4.8%) patients received <12 weeks of treatment, 258/315 (81.9%) received 12 weeks, 10/315 (3.2%) received >12 but <24 weeks, and 32/315 (10.2%) received 24 weeks. For patients without cirrhosis, these values were 9/191 (4.7%; <12 weeks), 170/191 (89.0%; 12 weeks), 5/191 (2.6%; >12 but <24 weeks), and 7/191 (3.7%; 24 weeks). For patients with cirrhosis, these values were 6/124 (4.8%; <12 weeks), 88/124 (71.0%; 12 weeks), 5/124 (4.0%; >12 but <24 weeks), and 25/124 (20.2%; 24 weeks).

Patient-completed survey results

Based on the first patient survey completed at the time of enrollment, English was the primary language for most (90.4%) patients. Less than 20% of patients reported consuming alcohol at least once per week and 34.0% were cigarette smokers. The majority of patients did not have a high degree of education (23.4% less than high school; 32.1% high school graduate or equivalent; and 22.4% some college, no degree). Total annual household income was less than $25,000 for more than half of patients. The diagnosis of HCV infection was most frequently made by a general physician, internal medicine physician, or primary care physician (55.4%). Most patients had no caregiver (77.8%) and did not participate in services or programs related to their HCV treatment (73.3%).

Based on the second patient survey completed posttreatment, most patients had no change in employment status (64.4%) and no change in their living situation (82.2%) compared with baseline. Only 18.1% of patients missed ≥1 medical appointment for HCV treatment during the study, and the most common reason for missing an appointment was lack of transportation and/or the patient could not get to the appointment (6.8%).

*Effectiveness*

One patient from the intent-to-treat (ITT) population was excluded from analysis of SVR12. This patient completed 25 weeks of treatment with simeprevir + sofosbuvir, and completed the study. HCV RNA was undetectable at Weeks 4 and 8, but was detectable 4 weeks after the last dose of study drug.

*Safety*

During the treatment phase, grade 3 (worst outcome) laboratory abnormalities were observed in ≤20% of patients with available data; the most common parameters (≥5% of patients) were elevated lipase (20.0%), hyperglycemia (9.5%), and increased platelets (5.4%). Grade 4 laboratory abnormalities were observed in ≤10% of patients; the most common (≥1% of patients) were elevated triacylglycerol lipase (10.0%) and elevated gamma-glutamyltransferase (4.2%).

**Supplemental Table 1. Subgroup Analysis of SVR12 Achievement in the mITT Population^a^**

| **Subgroup, n/N (SVR12 rate)^b^** | **Simeprevir + sofosbuvir**  **(n = 255)** | **Simeprevir + sofosbuvir + ribavirin**  **(n = 15)** | **Total**  **(N = 276)^c^** |
| --- | --- | --- | --- |
| Age, y |  |  |  |
| ≤45 | 22/24 (91.7) | 2/2 (100) | 24/27 (88.9) |
| >45-<65 | 170/187 (90.9) | 9/9 (100) | 182/200 (91.0) |
| ≥65 | 44/44 (100) | 4/4 (100) | 49/49 (100) |
| HCV genotype/subtype |  |  |  |
| 1a | 167/182 (91.8) | 11/11 (100) | 180/196 (91.8) |
| 1b | 52/55 (94.5) | 3/3 (100) | 56/60 (93.3) |
| Indeterminate | 17/18 (94.4) | 1/1 (100) | 19/20 (95.0) |
| Ethnicity^d,e^ |  |  |  |
| Hispanic/Latino | 35/41 (85.4) | – | 36/42 (85.7) |
| Not Hispanic/Latino | 197/210 (93.8) | 15/15 (100) | 215/230 (93.5) |
| Gender by race^d^ |  |  |  |
| Female |  |  |  |
| White | 50/54 (92.6) | 1/2 (50) | 52/57 (91.2) |
| Black/African American | 34/35 (97.1) | – | 34/35 (97.1) |
| Male |  |  |  |
| White | 89/96 (92.7) | 9/9 (100) | 101/108 (93.5) |
| Black/African American | 49/55 (89.1) | 5/5 (100) | 54/60 (90.0) |
| BMI, kg/m^2e^ |  |  |  |
| <25 | 62/68 (91.2) | 5/5 (100) | 67/73 (91.8) |
| ≥25-<30 | 76/78 (97.4) | 6/6 (100) | 85/89 (95.5) |
| ≥30 | 95/106 (89.6) | 4/4 (100) | 100/111 (90.1) |
| HIV co-infection |  |  |  |
| Yes | 17/22 (77.3) | – | 17/22 (77.3) |
| No | 219/233 (94.0) | 15/15 (100) | 238/254 (93.7) |
| Presence of cirrhosis |  |  |  |
| Yes | 91/100 (91.0) | 5/5 (100) | 98/107 (91.6) |
| No | 145/155 (93.5) | 10/10 (100) | 157/169 (92.9) |
| Prior HCV treatment history |  |  |  |
| Treatment-experienced | 65/72 (90.3) | 5/5 (100) | 70/77 (90.9) |
| Treatment-naïve | 171/183 (93.4) | 10/10 (100) | 185/199 (93.0) |
| Hepatic decompensation and simeprevir treatment duration (patients with cirrhosis)^e^ |  |  |  |
| Yes | 25/28 (89.3) | 1/1 (100) | 26/29 (89.7) |
| 12 weeks simeprevir treatment | 18/19 (94.7) | 1/1 (100) | 19/20 (95.0) |
| 12-<24 weeks simeprevir treatment | 1/1 (100) | – | 1/1 (100) |
| ≥24 weeks simeprevir treatment | 5/7 (71.4) | – | 5/7 (71.4) |
| Discontinued | 1/1 (100) | – | 1/1 (100) |
| No | 66/72 (91.7) | 4/4 (100) | 72/78 (92.3) |
| 12 weeks simeprevir treatment | 47/53 (88.7) | 3/3 (100) | 51/57 (89.5) |
| 12-<24 weeks simeprevir treatment | 3/3 (100) | – | 3/3 (100) |
| ≥24 weeks simeprevir treatment | 14/14 (100) | – | 15/15 (100) |
| Discontinued | 2/2 (100) | 1/1 (100) | 3/3 (100) |
| Calculated MELD score category^e^ |  |  |  |
| ≤10 | 78/83 (94.0) | 6/6 (100) | 84/89 (94.4) |
| ≥11-≤18 | 17/19 (89.5) | – | 17/19 (89.5) |
| ≥19-≤24 | – | – | – |
| ≥25 | 1/1 (100) | – | 1/1 (100) |
| Baseline employment status^e,f^ |  |  |  |
| Employed full time for wages | 45/49 (91.8) | 1/1 (100) | 46/50 (92.0) |
| Employed part time for wages | 10/11 (90.9) | – | 10/11 (90.9) |
| Self-employed | 12/14 (85.7) | – | 13/16 (81.3) |
| Not employed/unemployed | 19/20 (95.0) | 3/3 (100) | 22/23 (95.7) |
| Retired | 43/44 (97.7) | 5/5 (100) | 48/50 (96.0) |
| Short- or long-term disability | 45/49 (91.8) | 3/3 (100) | 49/53 (92.5) |
| Unable to work | 21/23 (91.3) | – | 22/24 (91.7) |
| Multiple | 17/19 (89.5) | 1/1 (100) | 18/20 (90.0) |
| Baseline living situation^e,f^ |  |  |  |
| Lived in 1 place the whole time | 208/225 (92.4) | 13/13 (100) | 224/243 (92.2) |
| Moved at least once during past year | 11/11 (100) | – | 11/11 (100) |
| Total annual household income (2013), USD^e,f^ |  |  |  |
| <$10,000 | 57/62 (91.9) | 4/4 (100) | 63/69 (91.3) |
| $10,000-$25,000 | 66/74 (89.2) | 5/5 (100) | 72/80 (90.0) |
| $25,001-$50,000 | 41/44 (93.2) | 3/3 (100) | 44/47 (93.6) |
| $50,001-$100,000 | 30/31 (96.8) | 2/2 (100) | 32/34 (94.1) |
| Out-of-pocket cost for all prescription medications/month^e,f^ |  |  |  |
| $0 | 47/51 (92.2) | 4/4 (100) | 52/57 (91.2) |
| $1-$25 | 75/80 (93.8) | 6/6 (100) | 83/89 (93.3) |
| $26-$50 | 40/46 (87.0) | – | 40/46 (87.0) |
| $51-$100 | 21/23 (91.3) | 3/3 (100) | 24/26 (92.3) |
| $101-$150 | 11/12 (91.7) | – | 11/12 (91.7) |
| RVR^e^ |  |  |  |
| Yes | 134/139 (96.4) | 11/11 (100) | 149/156 (95.5) |
| No | 69/81 (85.2) | 2/2 (100) | 71/83 (85.5) |
| Virologic response at the end of treatment^e,g^ |  |  |  |
| Yes | 210/223 (94.2) | 15/15 (100) | 229/244 (93.9) |
| No | 20/25 (80.0) | – | 20/25 (80.0) |

SVR12, sustained virologic response 12 weeks after the end of treatment; mITT, modified intent-to-treat; HCV, hepatitis C virus; BMI, body mass index; HIV, human immunodeficiency virus; MELD, Model for End-stage Liver Disease; USD, United States dollars; RVR, rapid virologic response at treatment Week 4; LLOQ, lower limit of quantification.

^a^SVR12 analysis was based on a snapshot approach. mITT population excludes patients who discontinued for non-virologic reasons before the SVR12 time point, or with missing SVR12 assessment data.

^b^Data are presented as n/N (SVR12 rate), where n is the number of patients in the subgroup who achieved SVR12, N is the total number of patients in the subgroup, and SVR12 rate is the percentage of patients who achieved SVR12.

^c^Includes 6 patients treated with simeprevir + peginterferon + ribavirin.

^d^Race and ethnicity data obtained from patients’ medical records.

^e^Not all patients had available data; only those with available data are included in the analyses.

^f^Only categories with ≥10 patients and a response are shown.

^g^Defined as HCV RNA undetectable at the end of treatment (excludes HCV RNA <LLOQ, but detectable).

**Supplemental Table 2. SVR12 Prognostic Factor Analysis for Patients in the mITT Population Treated With Simeprevir + Sofosbuvir^a^**

|  |  | **Univariate analysis^b^** | | **Multivariate analysis^b-d^** | |
| --- | --- | --- | --- | --- | --- |
| **Variable description** | **Categories included in model** | **Odds ratio (95% CI)** | ***P* value** | **Odds ratio (95% CI)** | ***P* value** |
| Gender | Female; male | 1.74  (0.60, 5.00) | 0.3069 | 2.69  (0.81, 8.92) | 0.1064 |
| Race^e^ | White; not White | 1.20  (0.46, 3.10) | 0.7117 | – | – |
| Ethnicity^e^ | Hispanic/Latino; not Hispanic/Latino | 0.32  (0.11, 0.92) | 0.0339 | 0.28  (0.09, 0.87) | 0.0283 |
| Age | Decades | 1.42  (0.96, 2.12) | 0.0811 | 1.49  (0.93, 2.39) | 0.0956 |
| BMI (kg/m²)/5^f^ | Continuous | 0.74  (0.52, 1.06) | 0.0997 | 0.64  (0.42, 0.96) | 0.0325 |
| HIV co-infection | Yes; no | 0.22  (0.07, 0.68) | 0.0090 | 0.15  (0.04, 0.55) | 0.0041 |
| Alcohol consumption^g^ | Yes; no | 1.71  (0.38, 7.74) | 0.4862 | – | – |
| Cirrhosis^h^ | Yes; no | 0.69  (0.27, 1.77) | 0.4386 | – | – |
| Hepatic decompensation | Yes; no | 0.72  (0.20, 2.66) | 0.6272 | – | – |
| HCV genotype/subtype | 1a; 1b or indeterminate | 0.62  (0.20, 1.94) | 0.4087 | – | – |
| Treatment history | Naïve; experienced | 1.59  (0.60, 4.25) | 0.3518 | – | – |
| Type of practice setting | Private practice clinic; other settings | 1.00  (0.36, 2.75) | 1.0000 | – | – |
| Number of patients seen per month | 1-20; 21-40; >40 | 0.88  (0.50, 1.55) | 0.6681 | – | – |

SVR12, sustained virologic response 12 weeks after the end of treatment; mITT, modified intent-to-treat; CI, confidence interval; BMI, body mass index; HIV, human immunodeficiency virus; HCV, hepatitis C virus.

^a^mITT population excludes patients who discontinued for non-virologic reasons before the SVR12 time point, or with missing SVR12 assessment data.

^b^Only patients with complete covariate information available were included (n = 228). Variables from the univariate analysis with *P* <0.90 were used in the multivariate analysis. Selection level to stay in the model (SLS) was 0.15, and a backward stepwise method was used in the multivariate analysis.

^c^Events/total = 209/228.

^d^C index = 0.773.

^e^Race and ethnicity data were from patients’ medical records.

^f^BMI values were divided by 5 to simplify interpretation of odds ratios.

^g^Alcohol consumption data (typical alcohol consumption) were from patient surveys completed at baseline.

^h^There were too few patients to include treatment duration in the model along with cirrhosis status (23/30 [77%] patients who received >12 weeks of treatment had cirrhosis, while 66/198 [33%] patients who received 12 weeks of treatment had cirrhosis); as such, treatment duration was not included in the models.

**Reference**

(1) World Health Organization. WHO toxicity grading scale for determining the severity of adverse events. Available at: <http://www.icssc.org/Documents/Resources/AEManual2003AppendicesFebruary_06_2003%20final.pdf>. Accessed May 18, 2016.
